# Vitamin K in Chronic Kidney Disease

**DOI:** 10.3390/nu11010168

**Published:** 2019-01-14

**Authors:** Mario Cozzolino, Michela Mangano, Andrea Galassi, Paola Ciceri, Piergiorgio Messa, Sagar Nigwekar

**Affiliations:** 1 Renal Division, Department of Health Sciences, University of Milan, 20142 Milan, Italy; michelamangano91@gmail.com (M.M.); andrea.galassi@asst-santipaolocarlo.it (A.G.); 2 Renal Research Laboratory, Department of Nephrology, Dialysis and Renal Transplant, Fondazione IRCCS Ca’ Granda, Ospedale Maggiore Policlinico & Fondazione D’Amico per la Ricerca sulle Malattie Renali, 20122 Milan, Italy; p.ciceri@hotmail.it (P.C.); piergiorgio.messa@unimi.it (P.M.); 3 Department of Medicine, Division of Nephrology, Massachusetts General Hospital, Boston, MA 02114, USA; SNIGWEKAR@mgh.harvard.edu

**Keywords:** vitamin K, CKD, vascular calcification

## Abstract

Vitamin K is a composite term referring to a group of fat-soluble vitamins that function as a cofactor for the enzyme γ-glutamyl carboxylase (GGCX), which activates a number of vitamin K-dependent proteins (VKDPs) involved in haemostasis and vascular and bone health. Accumulating evidence demonstrates that chronic kidney disease (CKD) patients suffer from subclinical vitamin K deficiency, suggesting that this represents a population at risk for the biological consequences of poor vitamin K status. This deficiency might be caused by exhaustion of vitamin K due to its high requirements by vitamin K-dependent proteins to inhibit calcification.

## 1. Introduction

There are two forms of vitamin K: K1 (phylloquinone, PK) mainly found in green vegetables, and K2 (including different menaquinones, MKs) derived from intestinal bacteria and fermented food (cheeses and “natty”, a Japanese soybean product) [1,2]. Liver is also a rich source of menaquinones [3]. We know about more than 12 different types of MKs, from MK-4 to MK-15, but the most common MKs in humans are the short-chain MK-4; it is the only MK produced by systemic conversion of phylloquinone to menaquinones [4].

Vitamin K is a non-polar molecule; after its intestinal absorption, vitamin k is solubilised by bile salt and pancreatic juice; vitamin K is then packaged into chylomicrons which are secreted into the lymphatic system [5]. For this reason lipids, in particular triglycerides, interfere with vitamin K measurement.

Vitamin K is as a substrate for an enzyme, the vitamin K-dependent carboxylase, that converts specific glutamic acid residues of a small number of proteins to glutamic carboxyl (Gla) residues by the addition of a CO_2_. Vitamin K is necessary to introduce carboxyl groups into glutamic acid residues in blood coagulation factors (II, VII, IX, X) to yield Gla residues [6]; Gla-containing proteins include osteocalcin (OC), synthesized by the osteoblasts in bone, and matrix Gla protein (MGP), synthesized by chondrocytes and vascular smooth muscle cells (VSMCs), involved in bone mineralization and inhibition of vascular calcifications, respectively.

## 2. Vitamin K Deficiency

Indirect functional tests can be assessed to detect vitamin k levels, such as the prothrombin time or measurement of undercarboxylated proteins OC and MGP, which are more sensitive in detecting subclinical vitamin K deficiency than prothrombin time [6]. This measurement is possible because vitamin K-dependent proteins (VKDP) cannot attain their carboxylation status (and they remain undercarboxylated) in the presence of vitamin K deficiency; this condition causes the loss of their capacity to bind calcium, so that bone metabolism may be impaired and the process of vascular calcification enhanced [7]. A high percentage of undercarboxylated OC (uOC) reflects vitamin K intake and the long-term vitamin K status. Osteocalcin levels are also influenced by vitamin D, which is required for the production of uOC, and by parathyroid hormone (PTH), which is frequently elevated in CKD patients [6,8,9]. Therefore, chronic kidney disease (CKD) patients with hyperparathyroidism will present high serum uOC, but this does not necessarily mean that they are vitamin K deficient.

A good alternative to evaluate vitamin K status is through the inactive form of MGP, even if inactive MGP may only reflect the vitamin K status at the vascular level and not at bone or liver. Indeed, randomized controlled studies have shown that vitamin K therapy decreases undercarboxylated unphosphorylated MGP (uc-dp-MGP) levels [9,10,11]; in contrast, anti-vitamin K (AVK) treatment increased the amount of inactive uc-dp-MGP [12] and stopping that treatment decreased uc-dp-MGP [13]. All these data suggest that uc-dp-MGP could be a good marker of vitamin K status [6].

## 3. Vitamin K Status in CKD Patients

Evidence demonstrates that CKD patients suffer from subclinical vitamin K deficiency [14], suggesting that this population is at risk for any consequences of poor vitamin K status. This deficiency might be caused by exhaustion of vitamin K due to its high requirement by vitamin K-dependent proteins to inhibit calcification. However, dietary recommendations for CKD patients, such as diets low in potassium (fewer leafy green vegetables rich in K1) and low in phosphate (fewer dairy products rich in K2) could promote this deficiency.

Considering that vitamin K is essential for the activation of matrix Gla protein (MGP), a powerful inhibitor of tissue calcification, functional vitamin K deficiency may contribute to high vascular calcification (VC) burden in haemodialysis patients; this is process in which mineral is pathologically deposited in blood vessels, mainly in large elastic and muscular arteries such as the aorta and the coronary, carotid, and peripheral arteries. The presence of VC is directly related to increased cardiovascular morbidity and mortality in dialysis patients [15]. 

Circulating non-phosphorylated non-carboxylated MGP, which is formed as a result of vitamin K deficiency in CKD, is associated with cardiovascular disease and with overall survival [16]; in contrast, in older individuals without CKD, non-carboxylated MGP was linked to abnormal vitamin K status, but not to coronary artery calcium content [17]. Shea et al. [17] reported the latter condition, and these findings are in contrast with results of case–control studies reporting higher plasma ucMGP among patients with diseases characterized by vascular calcification and kidney disease; however, the prior studies did not measure CAC directly as they have. Furthermore, the ucMGP may be a marker for other risk factors, such as hyperlipidaemia and hypertension [17].

Schurgers et al. measured dpucMGP in a cohort of 107 patients ranging from CKD stages 2–5D, and reported that levels of plasma dp-ucMGP increased progressively with CKD stage. They showed, therefore, a decline in this biomarker with reduction in GFR [18]; the risk of vascular calcification is presumed higher if the calcification-inhibitory effect of MGP is impaired. Pre-clinical studies confirm that diminished vitamin K status influences the severity of arterial calcification in rats with CKD that can be prevented by a diet rich in vitamin K1 [19].

Holden et al., in a study of 172 stage 3–5 CKD patients, demonstrated that over 50% consumed less than the recommended adequate intake for vitamin K; moreover, 6% were vitamin K-insufficient according to low levels of serum phylloquinone (<0.4 ng/mL), 60% were insufficient according to high levels of ucOC (>20% ucOC), and 97% were insufficient according to high levels of protein-induced vitamin K absence or antagonist II (PIVKA-II) (>2 nmol/L) [20].

Several studies focused on the vitamin K status in haemodialysis (HD) patients [21]. On account of its lipophilic characteristics, vitamin K would not be expected to be removed via the dialysis procedure but there is a growing and consistent body of evidence supporting the notion that maintenance HD patients have a global poor vitamin K status; while the exact reason for the low intake is unknown, it is likely related to the dietary regimen prescribed for HD patients and overall poor nutrient intake.

As reported by Westenfeld et al., at baseline, haemodialysis patients had 4.5-fold higher dephosphorylated, uncarboxylated MGP and 8.4-fold higher uncarboxylated osteocalcin levels compared with controls [11]. Cranenburg et al. measured and assessed vitamin K1 and K2 intake and the vitamin K status in 40 haemodialysis patients: the intake was low in these patients (median 140 µg/day), especially on days of dialysis and the weekend as compared to intakes reported in a reference population of healthy adults (mean K1 and K2 intake 200 and 31 µg/day, respectively) [14]. In response to poor dietary intake, body stores of vitamin K are rapidly depleted. Undercarboxylated vitamin K-dependent bone and coagulation proteins were found to be elevated in 33 haemodialysis patients, indicating subclinical hepatic vitamin K deficiency. Very high non-carboxylated MGP levels, endemic to all patients, also strongly suggested vascular vitamin K deficiency.

Voong et al. reported that 29% and 93% of maintenance HD patients met criteria for sub-clinical vitamin K deficiency based on low levels of phylloquinone and high levels of ucOC, suggesting there is also vitamin K deficiency at the level of bone [22].

Some studies demonstrated that peritoneal dialysis (PD) patients have a similar degree of vitamin K insufficiency as maintenance HD patients: in a study of 28 PD patients, 46% had vitamin K deficiency as measured by elevated PIVKA II levels (>2 nM/L) [23]. In a cross-sectional study of 21 PD patients, it was demonstrated that 24% of them had vitamin K deficiency as assessed by serum K1 (<0.4 nM/L) and all patients had vitamin K deficiency as assessed by increased percentage of ucOC (>20% was the cut off for vitamin K deficiency, mean was 60%) [24].

Jansz et al. studied the influence of kidney transplantation and phosphate binder use on vitamin K status and found that kidney transplant recipients have substantially lower dp-ucMGP levels compared to patients on haemo- or peritoneal dialysis, indicating better vitamin K status after restoration of kidney function. In general, instead, phosphate binder use is not associated with dp-ucMGP levels except for sevelamer monotherapy. which is associated with significantly higher dp-ucMGP levels, suggesting a negative effect of sevelamer on vitamin K status [25].

## 4. Anti-Vitamin K Treatment and Effects—Warfarin

Warfarin is a vitamin K antagonist; its use represents a model of vitamin K insufficiency. It induces arterial calcification in experimental models, but whether this occurs in humans is unclear. 

Tantisattamo et al. [26] examined breast arterial calcification identified on mammograms. Screening mammograms from women with current, past, or future warfarin use were examined for the presence of arterial calcification and compared with mammograms obtained in untreated women matched for age and diabetes mellitus. In 451 women with mammograms performed after ≥1 month of warfarin therapy, the prevalence of arterial calcification was 50% greater than in 451 untreated women (39.0% versus 25.9%; *p* < 0.0001). This study showed that the prevalence of breast arterial calcification is increased in women with current or past warfarin use independent of other risk factors and conditions predating warfarin use; this effect may be irreversible and appears to be cumulative.

McCabe at al. [19] wanted to determine whether modifying vitamin K status, either by increasing dietary vitamin K intake or by antagonism with therapeutic doses of warfarin, could alter the development of vascular calcification in male Sprague–Dawley rats with adenine-induced CKD. Pulse pressure and pulse wave velocity were increased after treatment with warfarin in CKD rats, as well as calcium concentrations in the thoracic aorta (3-fold), abdominal aorta (8-fold), renal artery (4-fold), and carotid artery (20-fold) were significantly increased. It is interesting to notice that therapeutic warfarin did not induce calcification in the blood vessels of healthy rats despite depletion of tissue vitamin K; in contrast, compared with CKD alone, warfarin markedly increased the levels of vascular calcification in all vessels studied in the presence of CKD. 

Thus, vitamin K has an important role in modifying mechanisms linked to the susceptibility of arteries to calcify in an experimental model of CKD.

As well as presenting a greater risk of development of vascular calcifications due to secondary hyperparathyroidism, haemodialysis patients are frequently treated with vitamin K antagonists, mainly to prevent stroke in atrial fibrillation, potentially compounding the cardiovascular risk in these already vulnerable patients. Warfarin may predispose to different side effect, such as bone fractures and vascular calcification, by different mechanisms: directly, by inhibition of carboxylation of OC and other bone matrix proteins; indirectly, because patients treated with warfarin may limit their dietary intake of foods rich in vitamin K [18,19]. 

### 4.1. Bone Fracture

Studies on bone fractures in CKD patients are limited. According to the available literature, the prevalence of vertebral fractures in dialysis patients appears to be similar to the general population, between 20.9% and 26.5% [27,28]; however Rodriguez-Garcia et al. [29] demonstrated a positive, significant correlation between vertebral fractures and vascular calcification in the dialysis population. This is probably due to underdiagnosis of vertebral fractures both in the general and in the dialysis population; this hypothesis was confirmed by the unpublished data of Fusaro et al., in which a vertebral fracture prevalence of 57% was found in a population of 68 haemodialysis patients [7]. Alem et al. studied the incidence of hip fractures and they found a considerably higher risk of hip fracture among dialysis patients than in general population [30].

Kohlmeier et al. were the first to demonstrate an independent association between poor vitamin K status and risk of bone fracture in patients in haemodialysis [31], while Fusaro et al. conducted an observational study of 387 prevalent HD, determining that over 50% of prevalent haemodialysis patients have vertebral fractures [32]. In this study, vitamin K1 deficiency (defined as a phylloquinone level adjusted for serum triglycerides <0.21 ng/mL), was the strongest predictor for the presence of a vertebral fracture (an adjusted odds ratio approaching 3).

In another study, Fusaro et al. found that haemodialysis patients treated with warfarin for greater than 1 year had an increased risk of vertebral fractures compared to those not on warfarin treatment [33]. With regard to vitamin K administration for the prevention of fractures, 13 randomized controlled studies with vitamin K1 and K2 treatment lasting for a minimum of 6 months were the subject of a meta-analysis [34]: 11 of these studies were from Japan, and seven reported fracture data. The subjects recruited in the studies were mainly menopausal women. All but one studies showed an advantage of vitamin K in reducing bone loss. All seven trials that reported fracture effects were from Japan and used vitamin K2 (menaquinone). Pooling the seven trials with fracture data in a meta-analysis, an odds ratio favouring menaquinone of 0.40 (95% confidence interval (CI), 0.25–0.65) for vertebral fractures, an OR of 0.23 (95% CI, 0.12–0.47) for hip fractures, and an OR of 0.19 (95% CI, 0.11–0.35) for all nonvertebral fractures were reported. 

### 4.2. Cardiovascular Calcification

The first study [35] demonstrating strongly increased calcification of the aortic valves in patients taking oral anticoagulants—including warfarin and acenocoumarol—has been confirmed by many other researchers: Koos et al. demonstrated that subjects taking vitamin K antagonist anticoagulants had a significantly higher degree of arterial and aortic valve calcification than control subjects [36]. In a case report, Hristova et al. described a kidney transplant recipient exhibiting massive arterial calcification after the initiation of warfarin therapy [37]. Distal subcutaneous necrosis ultimately led to the patient’s death. Rennenberg et al. showed rapid calcification of the femoral artery when patients are treated with oral anticoagulants, (OR 8.5; 95% confidence interval (CI) 2.01–35.95, for calcification in patients compared with controls) [38]. Similarly, patients taking oral anticoagulants showed significantly increased levels of coronary calcification [39].

### 4.3. Calciphylaxis

Approximately 1% of CKD patients develop calciphylaxis, a frequently fatal complication of end-stage renal disease. Notably, about 50% of patients with CKD stage 5D (CKD5D) who develop calciphylaxis are on vitamin K antagonists (VKAs) [40]. Compared to the dialysis patients worldwide, the overall number of calciphylaxis cases is minimal. However, in their case–control analysis, Hayashi et al. [41], calculated an 11.4-fold increased risk of developing calciphylaxis in Japanese dialysis patients on warfarin. 

In the pilot case–control study by Nigwekar et al. the authors demonstrated that patients with calciphylaxis in end stage renal disease (ESRD) had proportionally higher plasma levels of the inactive ucMGP than dialysis controls. They investigated how MGP carboxylation influenced the risk of calciphylaxis in 20 patients receiving haemodialysis with calciphylaxis (cases) and 20 patients receiving haemodialysis without calciphylaxis (controls); case patients had higher plasma levels of ucMGP and carboxylated MGP (cMGP) than controls. However, the fraction of total MGP that was carboxylated (relative cMGP concentration = cMGP/(cMGP + uncarboxylated MGP)) was lower in cases than in controls (0.58 ± 0.02 versus 0.69 ± 0.03, respectively; *p* = 0.003). The authors concluded that a vitamin K deficiency-mediated reduction in relative cMGP concentration may have a role in the pathogenesis of calciphylaxis [42].

Krueger et al. believe that vitamin K, and especially vitamin K2, supplementation may become an important option in the standard treatment for calcification-prone CKD patients [43].

### 4.4. Arteriovenous Fistula Failure

Arteriovenous fistula (AVFs) is a frequently used vascular access type for CKD patients requiring haemodialysis (HD) [44]. AVF failure is a complication leading to high hospitalization rates and morbidity [45]. Whereas early AVF failure is caused by thrombosis or by inability of the vein to dilate, later course AVF failure is induced by stenosis and thrombosis resulting from neointimal hyperplasia (NIH) and calcification [46,47]. Recent work indicates that AVF calcification contributes to AVF failure [48]; in arterialized veins, calcification occurs in tunica media and intima. HD patients have a poor overall vitamin K status, and a high number of CKD patients at risk of arterial and venous thrombosis receive oral anticoagulants (vitamin K antagonists; VKAs) [49]. It has been shown that VSMCs from varicose veins increased calcification when treated with warfarin, indicating similar processes in venous and arterial VSMCs [50]. Vascular access calcification is an independent predictor of mortality, whereas nothing is known about effects of VKA on AVF, NIH, and calcification.

The objective of the study of Zaragatski et al. was to investigate the effects of vitamin K antagonists and vitamin K2 treatment on neointimal hyperplasia development and calcification in rats and in arterialized human veins [51]. AVF was generated in female rats while chronic kidney disease was induced using an adenine-enriched diet. Arterialization, CKD, and vitamin K antagonists all significantly enhanced venous neointimal hyperplasia. K2 treatment and vitamin K antagonists significantly reduced neointimal hyperplasia in arterialized veins in healthy rats but not in rats with CKD. Arterialization, CKD, and vitamin K antagonism all significantly increased, whereas K2 supplementation attenuated calcification in healthy rats and rats with CKD.

K2 significantly enhanced matrix Gla protein carboxylation in control rats and rats with CKD. Arterialized human vein samples contained inactive matrix Gla protein at calcification and neointimal hyperplasia sites, indicating local vitamin K deficiency. Thus, vitamin K antagonists have detrimental effects on AVF remodeling, whereas K2 reduced neointimal hyperplasia and calcification indicating vasoprotective effects. Hence, K2 administration may be useful to prevent neointimal hyperplasia and calcification in arterialized veins.

## 5. New Oral Anticoagulant vs. Warfarin

New oral anticoagulants (NOACs) are valid alternatives to vitamin K antagonists in the general population, but their use in dialysis has been limited by substantial renal clearance.

Dabigatran and rivaroxaban have not been studied in end-stage renal disease patients who were specifically excluded from two large randomized trials that were conducted in patients without renal failure [52,53]. In dialysis patients, prescription of these novel oral anticoagulant drugs (NOACs) is currently contraindicated because the drugs are cleared by kidneys and drug levels can bio-accumulate to precipitate bleeding; as demonstrated by Chan et al., the unadjusted event rate of major bleeding increased with drug dosage [54]. The study of Chan et al. is the first to evaluate these drugs in the dialysis population, and suggests concern given the increasing use of dabigatran and rivaroxaban in the ESRD population despite formal FDA warnings of caution in renal failure. In fact, their secondary analyses suggest excess morbidity and mortality from bleeding associatively higher with dabigatran and rivaroxaban when compared to warfarin [54].

Among the available NOACs, apixaban has the lowest renal elimination. Both dabigatran and rivaroxaban were associated with increased bleeding in dialysis patients but recently apixaban was non-inferior to warfarin in a large database study.

Siontis et al. [55] conducted a retrospective cohort study in patients with ESRD and atrial fibrillation (AF) in dialysis who initiated treatment with apixaban or warfarin. Standard dose of apixaban (5 mg twice a day) was associated with significantly lower risks of stroke/systemic embolism and death as compared with either reduced dose of apixaban (2.5 mg twice a day; *n* = 1317; HR 0.61, 95% CI 0.37–0.98, *p* = 0.04 for stroke/systemic embolism; and HR 0.64, 95% CI 0.45–0.92, *p* = 0.01 for death) or warfarin (HR 0.64, 95% CI 0.42–0.97, *p* = 0.04 for stroke/systemic embolism; and HR 0.63, 95% CI 0.46–0.85, *p* = 0.003 for death). The authors concluded that, among ESRD patients with AF on dialysis, apixaban use may be associated with lower risk of major bleeding compared with warfarin, with a standard 5 mg twice a day dose also associated with reductions in thromboembolic and mortality risk.

## 6. Phosphate Binders

Chronic kidney disease mineral bone disorders (CKD-MBD) is a complex syndrome in dialysis patients, which includes alterations of biochemical parameters of the mineral and bone metabolism, a clinically and histologically manifested bone disease and vascular calcifications associated with an increased risk of cardiovascular morbidity and mortality. One of its most frequent components is hyperphosphatemia, which has traditionally been treated with dietary restrictions and intestinal phosphorus binders [56].

It is unknown if the use of phosphate binders, especially calcium based binders, may actually be harmful [57]. There is some attenuation or delay of progression of VC by the noncalcium-based phosphate binders as compared to the calcium containing binders [58,59,60]. However VC frequently progresses despite adequate phosphate concentrations; probably due to chronic inflammation, increasing calcium load from calcium based binders and other factors such as vitamin K deficiency.

In their study, Neradova et al. set up a study in which vitamin K2 (menaquinone-7; MK-7) was mixed with five different phosphate binders, in presence or absence of phosphate, incubated at pH 6 and fixed temperature of 37 degrees Celsius. In particular, 1 mg of vitamin K2 was added to a medium with pH 6 containing 67 mg phosphate binder with either 7 mg of phosphate or no phosphate [61].

This experiment showed that sucroferric-oxyhydroxide and sevelamer carbonate were the only binders that did not bind vitamin K2 in vitro. This in vitro study demonstrated that calcium acetate/magnesium carbonate binds vitamin K2 strongly both in absence (*p* = 0.001) and presence of phosphate (*p* = 0.003). For Lanthanum carbonate this binding depends on the absence of phosphate, pointing to competitive binding between phosphate and vitamin K2 for this compound (*p* = 0.005) whereas no significant binding of vitamin K2 was observed in the solution containing vitamin K2 and phosphate (*p* = 0.462). Calcium carbonate binds vitamin K2 statistically and significantly in a solution with vitamin K2 and phosphate (*p* = 0.009), whereas without phosphate no significant binding of vitamin K2 was observed (*p* = 0.123). In the mixture with sevelamer carbonate a nominally lower concentration of K2 was shown as well, but this decline was not statistically significant. Addition of sucroferric-oxyhydroxide did not lead to any decline of vitamin K2 at all, irrespective of presence or absence of phosphate.

The authors did not study the chemical explanation for this feature, but possibly the formed calcium-phosphate salt itself binds K2 as well. In conclusion, the presence or absence of phosphate significantly interferes with vitamin K2 binding, so phosphate binders could potentially limit the bioavailability of vitamin K2.

According to these results, in HD patients, in which phosphorus levels are usually elevated, phosphate binders that do not bind vitamin K in the presence of phosphate should be selected.

## 7. Drug and Vitamin K-Dependent Protein (VKDP)

Fusaro et al. carried out a sub-analysis to evaluate associations between drug consumption and VKDP levels in 387 haemodialyzed patients [62]. The VIKI study was an observational study performed in 387 adult patients of both genders who had been on dialysis for more than 1 year at 18 centres in Italy between November 2008 and November 2009. It was designed primarily to assess the prevalence of deficiency of vitamin K1 and vitamin K2 in dialysis patients. In this sub-study the authors evaluated drug consumption, determined bone Gla proteins (BGP) and MGP levels, and verified the presence of any vertebral fractures (VF) and VC by spine radiographs. Regarding mineral and bone disorder treatment, the most common were oral calcitriol (45.7%) and sevelamer (42.1%).

Total BGP levels were twice as high with calcimimetics versus no calcimimetics (290 vs. 158.5 mcg/L, *p* < 0.0001) and 69% higher with vitamin D analogues (268 vs. 159 mcg/L, *p* < 0.0001). Total MGP was 19% higher with calcimimetics (21.5 vs. 18.1 mcg/L, *p* = 0.04) and 54% higher with calcium acetate (27.9 vs. 18.1 mcg/L, *p* = 0.003); no difference was found with vitamin D analogues. This finding of increased MGP in patients treated with calcimimetics is the first in humans and it is consistent with the results of in vitro and in vivo experimental studies, in which MGP was measured before and after the administration of the calcimimetic [63,64,65].

Median Total BGP level was 29% lower in patients with ≥1 VF (151 vs. 213 mcg/L, *p* = 0.0091) and 36% lower in patients with VC (164 vs. 262.1 mcg/L, *p* = 0.0003). In non-survivors, median BGP and MGP were lower, but this difference reached the statistical significance for MGP only (152 vs. 191 mcg/L, *p* = 0.20 and 15.0 vs. 19.7 mcg/L, *p* = 0.02, respectively). No significant association between MGP levels and VF or VC was found, despite an association between high MGP levels and survival.

Pending studies on vitamin K supplementation, calcimimetics, and vitamin D analogues may play a role in preserving vitamin K-dependent protein activity, thus contributing to bone and vascular health in CKD patients.

## 8. Vitamin K Supplementation

The question therefore arises of whether CKD patients might benefit from extra vitamin K intake. In the population-based Rotterdam study [66], dietary intake data including vitamin K were available for 4800 elderly patients. The authors assessed the risk of incident coronary heart disease, all-cause mortality, and aortic calcification based on tertiles of energy-adjusted vitamin K intake. They found that low vitamin K2 intake was associated with a higher incidence of severe aortic calcification and increased mortality. In line with this observation, a recent pilot study investigated whether daily vitamin K2 supplementation would improve the bioactivity of vitamin K-dependent proteins in CKD5D patients: after only 6 weeks of daily vitamin K2 supplementation, a significant decrease in dp-uc-MGP, ucOC, and uncarboxylated factor II (ucFII) levels was observed. This shows that vitamin K is able to reach tissues, including the vessel wall, and to correct the biochemical and local tissue consequences of vitamin K deficiency.

A supplementation in vitamin K in particular in HD an PD patients should be taken into account, considering the poor vitamin K status in CKD patients. One randomized controlled trial evaluated the response of biomarkers of vitamin K status (dp-uc-MGP, PIVKA-II and ucOC) to three doses of vitamin K2 (MK-7) administered over a period of 6 weeks in HD patients and the study reported that vitamin K2 (MK-7) supplementation decreased dp-uc-MGP and PIVKA-II levels [11]. Shea et al. reported that supplementation with daily phylloquinone (0.5 mg) slowed the progression of cardiovascular calcification (CAC) over 3 years in healthy older adults with pre-existing CAC at baseline [67]; it is well established that patients with kidney failure have an increased risk for both vascular calcification and sub-clinical vitamin K deficiency but yet no trial that examines whether vitamin K supplementation prevents the progression of calcification in this population has been completed.

Furthermore, vitamin K status after kidney transplantation may play an important role. In a study made by Keyzer et al. [68], vitamin K deficiency was an important risk factor of overall mortality in kidney-transplanted patients.
